# Risk and Spatial Spread of a Measles Outbreak in Texas

**DOI:** 10.3390/v18060648

**Published:** 2026-06-04

**Authors:** Martial Loth Ndeffo-Mbah, Sina Mokhtar, Abhishek Pandey, Chad Richard Wells

**Affiliations:** 1 Department of Epidemiology & Biostatistics, Texas A&M University, College Station, TX 77843, USA; 2 Department of Veterinary Integrative Biosciences, Texas A&M University, College Station, TX 77843, USA; 3 Department of Mathematics & Statistics, University of New Mexico, Albuquerque, NM 87106, USA; 4 Center for Infectious Disease Modeling and Analysis, Yale University, New Haven, CT 06520, USA

**Keywords:** measles outbreak, spatial spread, MMR vaccination, epidemic modeling, Texas

## Abstract

In January 2025, a measles outbreak was reported in Gaines County, Texas, and subsequently spread to other counties and states. However, investigations into the geographic spread of this outbreak remain limited. We developed a measles transmission model parameterized with 2020–2024 measles–mumps–rubella (MMR) vaccination coverage and human mobility data in Texas. We conducted sensitivity analyses to evaluate how variation in model parameters affects outcomes. We compared model predictions to data from the 2025 measles outbreak and simulated scenarios for outbreak originating in different low vaccination counties. We found that an outbreak originating in Gaines County would have at least 80% probability of directly generating local outbreaks in eight neighboring counties in West Texas. The spatial spread was highly sensitive to the basic reproduction number ($R_{0}$) and population-level MMR vaccination coverage. Outbreaks originating in counties with low MMR vaccination rates, such as Polk, Montague, or Limestone County, are likely to spread to large metropolitan areas such as Houston and Dallas. Measles has the potential to cause statewide outbreaks in Texas. Our modeling framework can inform county-level risk assessment and guide preemptive vaccination strategies.

## 1. Introduction

Measles is an acute, highly contagious respiratory viral disease that typically presents with a blotchy skin rash. The virus infects around 90% of non-immunized individuals exposed to an infectious case [1]. Though measles usually does not cause long-term medical issues, infection can cause severe complications and even death [2]. Following the isolation of the measles virus in 1954, the first measles vaccine was licensed in the United States of America (USA) in 1963 [3,4]. Since then, the measles vaccine has undergone refinement, leading to the highly effective and safe measles, mumps, and rubella (MMR) vaccine widely used today [3]. Measles vaccination has dramatically reduced the burden of measles in the United States and globally [5].

In the USA, measles was declared eliminated in 2000, a testament to the vaccine’s success [3]. However, measles outbreaks continue resurfacing in the USA [6]. The country has witnessed sporadic outbreaks in the post-elimination period, often linked to international travel. These outbreaks highlight the importance of sustained vaccination efforts and vigilant surveillance. According to the US Centers for Disease Control and Prevention (CDC), between 2001 and 2019, 158 measles outbreaks were reported in the US, resulting in over 3000 cases [6]. Most of these outbreaks occurred in communities with low vaccination rates, underscoring the critical role of herd immunity in preventing measles outbreaks.

On 23 January 2025, the Texas Department of State Health Services (DSHS) reported the first measles cases in the state since 2023 [7]. On 29 January, local transmission among unvaccinated school-aged children was identified in Gaines County, Texas [7]. The outbreak originated in a predominantly Mennonite community with low vaccine uptake. By 4 March, 159 cases were reported in Texas, with 107 in Gaines County alone. Since then, the outbreak has spread throughout West Texas and beyond, with Gaines County serving as the epicenter. By 12 August, 762 cases had been reported in 24 counties out of the 245 counties in Texas, with 54.3% of cases in Gaines County [8]. In response to this ongoing outbreak, DSHS launched communications, testing, and vaccination campaign efforts to mitigate the spread and burden of measles in Texas.

In this study, we developed a data-driven model to estimate the risk of spatial spread of a measles outbreak in Texas at the county-level. Our model integrates county-level school immunization data from Texas DSHS and a human mobility dataset derived from anonymized mobile phone records (SafeGraph). Though we used the most up-to-date open access mobility and immunization data to parameterize our model, our study objective is to propose a modeling framework to estimate the county-level risk of spatial spread of a measles outbreak in Texas rather than performing a retrospective analysis of the 2025 measles outbreak in Texas. To assess the sensitivity of our model outcomes to input parameters, we conducted several one-way sensitivity analyses by varying the value of the key input parameters over a realistic range of values.

## 2. Materials and Methods

### 2.1. Transmission Risk Model

To estimate the risk of a measles outbreak spreading in Texas, we considered the state as a network of counties linked through human mobility. The basis of the model is that the network of contacts between counties can provide estimates of the probability that a county outbreak can seed an outbreak in an interconnected county. For each pair of counties, we calculated the probability that an outbreak could be seeded in county *i* given that an outbreak occurs in neighbouring county *j*.

The probability that an outbreak in a given county (source county) leads to an outbreak in another county (receiving county) through a single contact is a function of three independent probabilities: (1) the probability that an individual in the source county gets infected during the outbreak in the source county, (2) the probability that an individual in the receiving county is susceptible to measles infection, and (3) the probability that a receiving county will experience an outbreak given that one of its resident becomes infected. The probability that an individual in county *j* becomes infected during a local outbreak is denoted by $P_{j}^{I}\;$, and the probability that an individual in county *i* is susceptible is denoted by $P_{i}^{S}\;$. The probability that a single infected individual in county *i* causes a large outbreak in that county is denoted by $P_{i}^{LO}$. So, the probability that an outbreak in county *j* leads to an outbreak in county *i* through a single contact between the counties is given by $P_{j}^{I}P_{i}^{S}P_{i}^{LO}$.

We assumed $P_{j}^{I}\;$ to be equal to the proportion of individuals infected during the outbreak in county *j*. This proportion is considered to be equal to the solution of the final outbreak size equation [9,10]

$$P_{j}^{I}=(1-M_{j})\left(1-e^{-R_{0}P_{j}^{I}}\right)$$

where $R_{0}$ is the measles basic reproduction number, and $M_{j}$ is the immunity level in county *j*. The basic reproduction number is defined as the number of secondary cases caused by a single infective case in a completely susceptible population [11].

We assumed that $P_{i}^{S}$ is equal to $\left(1-M_{i}\right)$, which is the proportion of the population unimmunized (unvaccinated or not effectively immunized). We defined $P_{i}^{LO}$ using the Anderson & Watson formula for the probability of a major outbreak of an SEIR-type pathogen in a susceptible population [12,13].

$$P_{i}^{LO}=1-\frac{1}{(1-M_{i})R_{0}}$$

Here, a major outbreak refers to a continuous chain of pathogen transmission with a reproduction number greater than 1 [12,13]. An SEIR-type pathogen is a pathogen, such as the measles virus, whose outbreak dynamics can be modeled using an SEIR (Susceptible-Exposed-Infectious-Recovered) model [14,15].

We set $R_{0}$ to be equal to 18, which is the frequently reported value of measles $R_{0}$ [11]. Given that county-level immunization data across all age groups are not available, we assume that the measles immunity level of a given county $M_{i}$ is directly related to the proportion of vaccinated children younger than 10 years old [16]. We set $M_{i}=\epsilon V_{i}$ where ϵ the MMR vaccine efficacy is equal to 0.97 [17], and $V_{i}$ is the proportion of vaccinated children younger than 10 years old set to be equal to the average MMR vaccination coverage among kindergarteners from 2020–2024 (Figure S1) [18]. Information on county name, school-level MMR vaccination coverage, and geographical location are available in Figure S1.

If $C_{ij}\;$ is the number of contact pairs that link county *i* and *j*, the probability that at least one contact pair causes a major outbreak in county *i* is given

$$P_{ij}=1-{\left(1-\left(P_{j}^{I}P_{i}^{S}P_{i}^{LO}\right)\right)}^{C_{ij}}.$$

**Figure 1:**
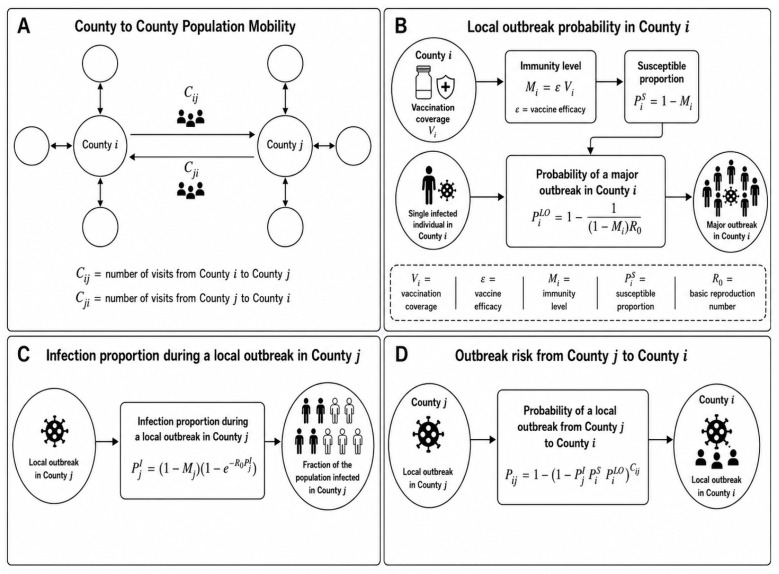
Schematic diagram of the model. (**A**) A bidirectional network of counties constructed such that counties are connected by the number of visits (mobility flow) from one county to another. (**B**) The probability that a county experienced a large outbreak caused by a single new infected individual. (**C**) Measles attack rate in a county that experienced an outbreak. (**D**) The probability that at least one contact pair between a source county (experiencing an outbreak) and a receiving county (outbreak free) causes a major outbreak in the receiving county.

We defined $C_{ij}$ as the number of visits from county *i* to county *j* during 8 months (mid-January to mid-August, corresponding to the duration of the largest measles outbreak in Texas since 2000 [8]). To estimate $C_{ij}$, we used between-county visitor flows in 2019 computed from anonymous mobile phone users’ visit data provided by SafeGraph (Denver, CO, USA) [19]. The flows dataset was validated against the American Community Survey commuting dataset [19]. Model’s parameters, baseline values, and sources are summarized in Table 1. A schematic description of our model is provided in Figure 1.

A county outbreak resulting from contacts with infected individuals from the source county (e.g., Gaines County) would be denoted as a first-generation outbreak. Though the model can be extended to account for higher-order generations such as second- and third-generation outbreaks, we limited our model to first-generation outbreaks given that the bulk of measles cases and active transmission events, in the recent large-scale measles outbreak in Texas, have been limited to a handful of counties (Table S1).

### 2.2. Sensitivity Analysis

To assess the sensitivity of our model outcomes to the input parameters value, we conducted one-way sensitivity analysis on the value of $R_{0}$, county-level vaccination coverage, and between counties mobility intensity. These three parameters are paramount to our modeling framework. To evaluate the sensitivity of our results to the underlying MMR vaccination coverage, we consider two scenarios where the county-level MMR coverage is 5% lower than the school-level MMR coverage and 5% higher than the school-level MMR coverage. This allowed us to consider a wide range of value for the county-level MMR coverage relative to the school-level vaccination coverage. To evaluate the sensitivity of our results to the underlying measles transmission risk, we consider $R_{0}=\;12$, and 16, where $R_{0}=16$ is the median value of measles $R_{0}$ in least developed countries during the MMR vaccination era [20], and $R_{0}=12$ is the median measles $R_{0}$ value during the vaccination era in developed countries [20]. Finally, we evaluate the sensitivity of our results to the underlying mobility intensity between counties by increasing and reducing the baseline 2029 mobility rate by 5% and 10% which is consistent with observation from an empirical study comparing within-city human mobility from in- and out-of-state movement during pre- and post-pandemic periods [21].

### 2.3. Alternative Outbreak Scenarios

We considered alternative scenarios where a hypothetical 2025 measles outbreak in Texas originated in other Texas counties. We focused on outbreaks starting in counties with low school-level vaccination coverage and compared their risk of spatial spread throughout the state to an outbreak originating in Gaines County. We specifically focused on King County, Hall County, and Throckmorton County, which were identified as the lowest vaccination coverage counties in Texas, with 80.6%, 82.0%, and 83.4% coverage, respectively. These three counties are sparsely populated, low-population-density rural counties, isolated from large Texas metropolitan areas. In addition, we considered low vaccination coverage counties such as Limestone County, Montague County, and Polk County, with 86.4%, 86.8%, and 89.2% coverage, respectively. These counties are situated in proximity to the largest metropolitan areas, which are Houston and Dallas-Fort Worth.

## 3. Results

**Figure 2:**
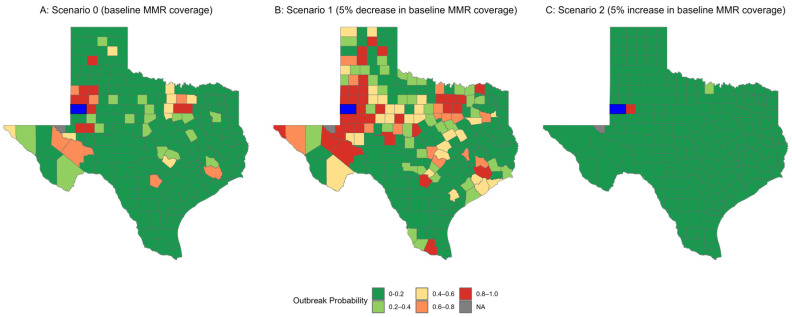
Probability that a measles outbreak originating in Gaines County leads to major outbreaks in other Texas counties. Gaines County, the source of the outbreak, is denoted in blue. Here, all model parameters were kept at their Table 1 value, except indicated otherwise. (**A**) Outbreak probability under baseline MMR coverage in each county. (**B**) Outbreak probability under a 5% decrease in baseline MMR coverage in each county. (**C**) Outbreak probability under a 5% increase in baseline MMR coverage in each county. The county shapefile was obtained from the US Census Bureau.

Our simulations show that under our baseline parameters value (Table 1), a 2025 measles outbreak originating in Gaines County would likely spread to neighbouring counties and generate intra-county outbreaks (large-scale local transmission) mostly in West Texas (Figure 2). West Texas counties such as Lubbock, Terry, Yoakum, Dawson, Ector, Randall, Hockley, and Midland had a high probability (above 0.8) of experiencing a local outbreak directly initiated from the Gaines County outbreak. In contrast, Cochran, Reeves, and El Paso County had a moderate risk, around 0.5 (Figure 2A). To evaluate the impact of the county-level MMR coverage on the model results, we assume that the county-level immunity was either 5% lower than the school-level MMR coverage or 5% higher than the school-level MMR coverage. If county-level immunity was 5% lower than the school-level MMR coverage in each county, the probability that a measles outbreak originating in Gaines County leads to major outbreaks in another Texas county was substantially increased for many counties (Figure 2B). However, if county-level immunity was greater than the school-level MMR coverage, the Gaines outbreak only spread to a single county (Figure 2C).

**Figure 3:**
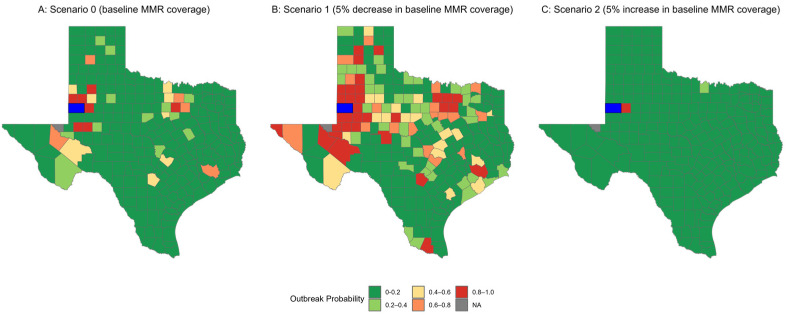
Probability that a measles outbreak originating in Gaines County leads to major outbreaks in other Texas counties. Gaines County, the source of the outbreak, is denoted in blue. Here, all model parameters were kept at their Table 1 value, except $R_{0}=16$. (**A**) Outbreak probability under baseline MMR coverage in each county. (**B**) Outbreak probability under a 5% decrease in baseline MMR coverage in each county. (**C**) Outbreak probability under a 5% increase in baseline MMR coverage in each county. The county shapefile was obtained from the US Census Bureau.

**Figure 4:**
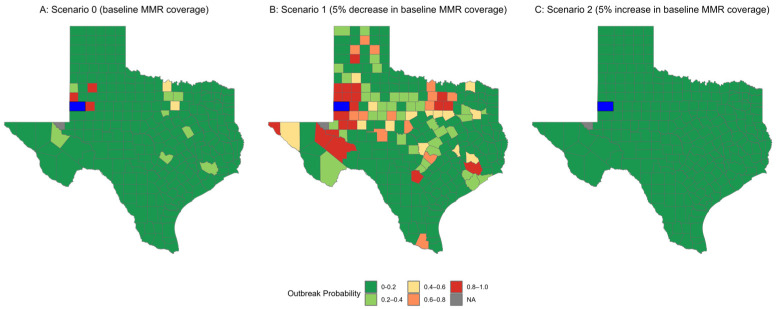
Probability that a measles outbreak originating in Gaines County leads to major outbreaks in other Texas counties. Gaines County, the source of the outbreak, is denoted in blue. Here, all model parameters were kept at their Table 1 value, except $R_{0}=12$. (**A**) Outbreak probability under baseline MMR coverage in each county. (**B**) Outbreak probability under a 5% decrease in baseline MMR coverage in each county. (**C**) Outbreak probability under a 5% increase in baseline MMR coverage in each county. The county shapefile was obtained from the US Census Bureau.

To evaluate the impact of measles $R_{0}$ value on our model results, we consider two alternative values: $R_{0}=16$, the median measles $R_{0}$ value in least developed during the MMR vaccination era [20], and $R_{0}=12$, the median measles $R_{0}$ value in developed during the MMR vaccination era [20]. When $R_{0}=16$, the risk of spatial spread of a measles outbreak originating in Gaines County (Figure 3) was shown to be marginally different from the risk under the frequently reported $R_{0}$ value of 18 (Figure 2). However, substantial difference in outbreak risk was observed when $R_{0}=12$ (Figure 4). In all scenarios, a measles outbreak originating in Gaines County was shown to spread mostly within West Texas.

These results on spatial spread are consistent with reported measles cases of the 2025 measles outbreak in West Texas. Of the 12 Texas counties that had reported six or more measles cases (twice the CDC’s minimal measles outbreak size [22]) from the outbreak originating in Gaines County, our model showed that 11 of them have more than a 0.5 probability of experiencing an outbreak, especially when county-level immunity was assumed to be 5% lower than its corresponding school-level MMR coverage (Figure 2, Figure 3 and Figure 4 and Table S1).

To evaluate the impact of mobility intensity on our model results, we vary the value of the baseline mobility flow between counties, the 2019 empirical mobility data, by ±5% and ±10% (Figures S2–S7). For each $R_{0}$ value the outbreak probability was shown to be marginally impacted by these changes in mobility (Figures S2–S7).

**Figure 5:**
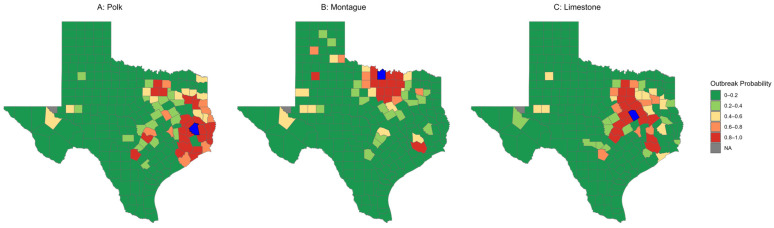
Probability that a measles outbreak originating in Polk, Montague, and Limestone counties leads to major outbreaks in other counties. (**A**) Outbreak originating in Polk County; Polk County is denoted in blue. (**B**) Outbreak originating in Montague County; Montague County is denoted in blue. (**C**) Outbreak originating in Limestone County; Limestone County is denoted in blue.

King County (80.6%), Gaines County (81.7%), Hall County (82.0%), and Throckmorton County (83.4%) were identified as the counties with the lowest county-level MMR vaccination coverage (Figure S1). We investigated the spatial spread of a hypothetical measles outbreak originating in King, Hall, or Throckmorton County. We showed that an outbreak originating in King County is unlikely to spread widely to other Texas counties through first- or second-generation outbreaks (Figure S8A). This was likely due to its remote location and limited interaction (number of between-county visits) with other counties. An outbreak originating in Throckmorton County was shown to have a high probability of spreading to other North Texas counties (Figure S8C), whereas an outbreak originating in Hall County is likely to spread statewide (Figure S8B). In addition to these counties with the lowest MMR coverage, we considered other low vaccination coverage counties, such as Limestone, Montague, and Polk County (Figure 5). These counties are situated in proximity to large metropolitan areas such as Dallas-Fort Worth and Houston. We investigated the spatial spread of outbreaks originating in these counties and showed that such outbreaks would have high probability of spreading widely and causing local major outbreaks in densely populated areas such as the Dallas-Houston Corridor area and Travis and Bexar counties (Figure 5).

## 4. Discussion

We estimated the risk of the spatial spread of a 2025 measles outbreak in Gaines County, Texas. Though such an outbreak can spread beyond state borders, our analysis was limited to the spread across Texas counties. Using county-level MMR vaccination coverage from 2020 to 2024, and empirical data on between-county mobility, we showed that a measles outbreak originating in Gaines County would have at least an 80% probability of directly generating local outbreaks in eight neighbouring counties in West Texas (Lubbock, Terry, Yoakum, Dawson, Ector, Randall, Hockley, and Midland County). These county-level outbreaks resulting from direct contact with infected residents of Gaines County were denoted as first-generation outbreaks. We conducted sensitivity analysis on key input parameters such as the basic reproduction number, mobility intensity, and vaccination coverage. We showed that the risk of spatial spread of a measles outbreak is very sensitive to the underlying population-level immunity across counties and the measles $R_{0}$ value during the outbreak.

As increased vaccination coverage is the most effective approach for preventing and curtailing the spread of measles, recent modeling studies have investigated the potential impact of vaccination on the risk of measles outbreaks in the US [15,23,24,25]. MMR vaccination coverage is highly heterogeneous across Texas, with several county-level coverages below 90% [18]. Counties with low MMR vaccination coverage are regarded as hotspots for measles outbreaks [26,27]. Though the recent West Texas measles outbreak, which originated in Gaines County, is one of the largest outbreaks in the measles post-elimination era, our model showed that measles outbreaks in other low vaccination counties, such as Polk, Limestone, and Montague County, could quickly spread to densely populated counties and potentially affect more counties than the 2025 West Texas outbreak. Our results highlight a synergistic contribution of vaccination coverage and human mobility to the risk of geographical spread of a measles outbreak in Texas. This result agrees with previous studies showing that human mobility plays a key role in the spread of emerging and re-emerging infectious diseases [28,29,30].

Our modeling framework estimates the probability of a major outbreak as its main outcome measure. This measure accounts for the potential impact of epidemiological uncertainties in infectious disease transmissions. A county is identified as a high-risk county if it has a high probability of experiencing a major local outbreak. Such a high probability indicates low vaccination coverage and a high risk of case importations due to high contact volume with other high-risk counties. Conversely, a low likelihood of experiencing a major outbreak does not indicate that a county may not report several measles cases during a statewide outbreak. Here, reported measles cases in low-risk counties may simply be imported cases (infection acquired out-of-county) or the result of small outbreaks with limited transmission events [31,32].

Our modeling approach differs from prior spatial transmission modeling studies of measles in the U.S. [33,34,35,36,37]. We use a probabilistic network framework parameterized with empirical mobility data and county-level vaccination coverage to directly estimate pairwise outbreak importation risk, rather than simulating full epidemic dynamics. This approach makes our model easy-to-use and less computationally and epidemiologically demanding than earlier modeling studies in the United States that typically relied on deterministic or stochastic SEIR-type models calibrated to outbreak time series [33,34,35,36,37]. These models incorporated space through metapopulation structures and standard mobility models, focusing on transmission dynamics, intervention effects, or outbreak size. While these studies highlighted the role of vaccination heterogeneity and clustering [33,34,35,36,37], ours explicitly integrate mobility-driven connectivity with immunity heterogeneity to generate forward-looking, county-level risk maps, and emphasize probabilities of major outbreaks rather than case trajectories, offering a more scalable but less temporally detailed framework.

Like other modeling studies, our study has several limitations, mostly related to data availability and model assumptions. First, our model used 2019 mobility data on between-county visits, which was informed by the 2019 anonymous mobile phone users’ visits data provided by SafeGraph. To the best of our knowledge, the 2019 SafeGraph mobility data is the most up-to-date open access mobility dataset that provides large-scale empirical data on between county mobility in Texas. These data provide a more accurate description of population mobility than standard mobility models, such as the gravity and radiation models [38]. Using prior COVID pandemic mobility data can be regarded as a good approximation of the post-pandemic mobility. Though using post-COVID pandemic mobility data may have been ideal to inform 2025 mobility, such data are not freely available. To assess sensitivity to mobility intensity, we evaluated how increases and decreases in mobility relative to baseline levels affected our results. Our analysis shows that modest changes in mobility intensity have a marginal impact on the risk of measles importation in each county. However, a substantial change in mobility, as observed during the pandemic, is likely to substantially impact the outbreak risk as observed with respiratory diseases incidence during the COVID-19 pandemic [21]. Second, our model uses elementary school vaccination data to inform the county-level immunization rate, as county-level MMR immunization data in Texas are not publicly available. Though most measles cases in the US occur among school-age children [27], assuming that the county vaccination rate is proportional to its average coverage among elementary school students ignores potential variation in immunization rates among older children and adults, as well as the within-county spatial clustering of non-vaccinators that may enhance the risk of local outbreaks [39,40]. We conducted a sensitivity analysis on county-level MMR coverage by varying the baseline school-level coverage by ±5%. This analysis showed that the risk of spatial spread of a measles outbreak in Texas is highly sensitive to the true immunity-level of each county. Future work should investigate population-level immunity at the county-level in Texas. The estimate of population-level immunity against measles should incorporate maternal immunity, immunity among 1- to 4-year-olds resulting from 1- and 2-dose induced immunity, school-age immunization rate, immunity among young adults (utilizing historical vaccination coverage and vaccine waning), and immunity among adults and the elderly. Such refined estimates of population-level estimates of measles immunity would greatly improve the estimation of outbreak risk. Finally, our approach does not account for transient dynamics in outbreak risk, which may be important for forecasting infection cases and hospitalizations. To explicitly account for transient epidemic dynamics, our model can be extended to incorporate within-county/city/school-district disease transmission dynamic models. Calibrating such models would require county/city/district-level time series data on measles cases. However, these time series data are not generally publicly available at the right spatial resolution. Our modeling framework aims only to estimate the county-level spatial spread of a measles outbreak in Texas. Although its utility may be limited, it provides a simple, data-driven, and easy-to-use framework for identifying counties at risk of experiencing an outbreak initiated by a source county. This is crucial for allocating resources to at-risk counties and developing strategies to mitigate the spatial spread of an outbreak. Employing this framework will require the use of updated data, such as mobility and MMR vaccination coverage.

## 5. Conclusions

Our results indicate that measles remains a major public health problem in Texas, with the potential risk of causing large-scale statewide outbreaks. This risk is exacerbated by the recent decline in MMR vaccination in the US since the peak of the COVID pandemic [41]. If vaccination rates continue to decline, large-scale measles outbreaks are likely to become frequent in the US in general and in Texas in particular. Our study provides a simple and easy-to-use data-driven modeling framework that can be used to estimate the county-level risk and geographical spread of a measles outbreak. This modeling framework is easily applicable to other US states and can be used at higher spatial resolutions, such as school-district or city-level, conditioned on the availability of vaccination and mobility data.

## Figures and Tables

**Table 1:** Model parameters.

Parameter	Description	Values	Source
$R_{0}$	Basic reproduction number	18	[11]
$V_{i}$	MMR vaccination coverage in county *i*	Varies between county	County-level MMR vaccine uptake in elementary school from Texas DSHS [18]
ϵ	Vaccine efficacy	0.97	[17]
$C_{ij}$	The number of visits from county *i* to county *j*	Varies between counties	SafeGraph mobility data [19]

## Data Availability

No additional data available. All codes are available at https://github.com/30-na/Measles_Outbreak_Spread_Risk (3 June 2026).

## Supplementary Materials

The following supporting information can be downloaded at: https://www.mdpi.com/article/10.3390/v18060648/s1. Figure S1: County-level school MMR vaccination coverage used as baseline county-level immunity in our analysis. Figure S2: Sensitivity analysis of the probability that a measles outbreak originating in Gaines County leads to major outbreaks in other Texas counties. Figure S3: Sensitivity analysis of the probability that a measles outbreak originating in Gaines County leads to major outbreaks in other Texas counties. Figure S4: Sensitivity analysis of the probability that a measles outbreak originating in Gaines County leads to major outbreaks in other Texas counties. Figure S5: Sensitivity analysis of the probability that a measles outbreak originating in Gaines County leads to major outbreaks in other Texas counties. Figure S6: Sensitivity analysis of the probability that a measles outbreak originating in Gaines County leads to major outbreaks in other Texas counties. Figure S7: Sensitivity analysis of the probability that a measles outbreak originating in Gaines County leads to major outbreaks in other Texas counties. Figure S8: Probability that a measles outbreak originating in King, Hall, and Throckmorton County leads to a major outbreak in other counties. Table S1: Outbreak cases by county in West Texas as of 12 August 2025.
