# Supplementary material for: Risk and Spatial Spread of a Measles Outbreak in Texas
Source: Viruses. 2026 Jun 4;18(6):648. doi: 10.3390/v18060648 (PMC13307820; doi:10.3390/v18060648)
Supplement: Supplementary file 1 [file viruses-18-00648-s001.zip › viruses-4342202-supplementary.pdf]

Supplementary Materials

1 Supplementary Figures

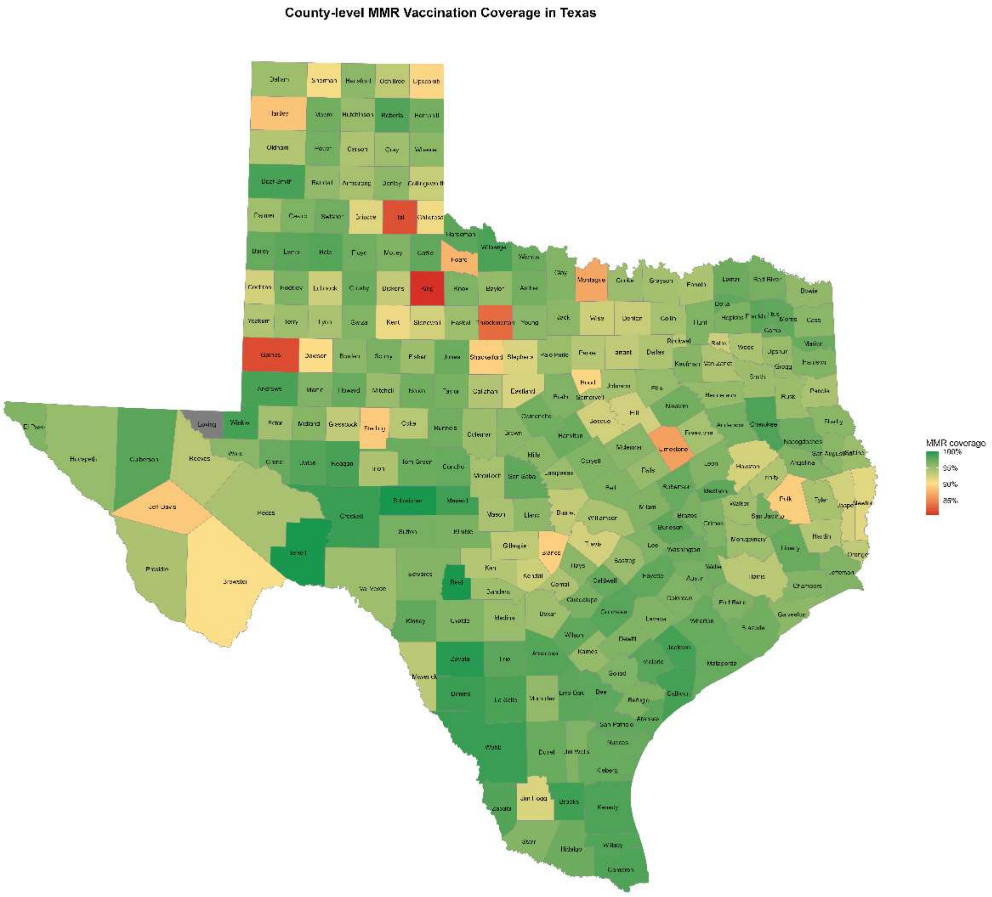

Supplementary Figure S1: County-level school MMR vaccination coverage used as baseline county-level immunity in our analysis. For each county, the map shows county name, geographical location, and the average county-level vaccination coverage among kindergartners in Texas from 2020 to 2024.

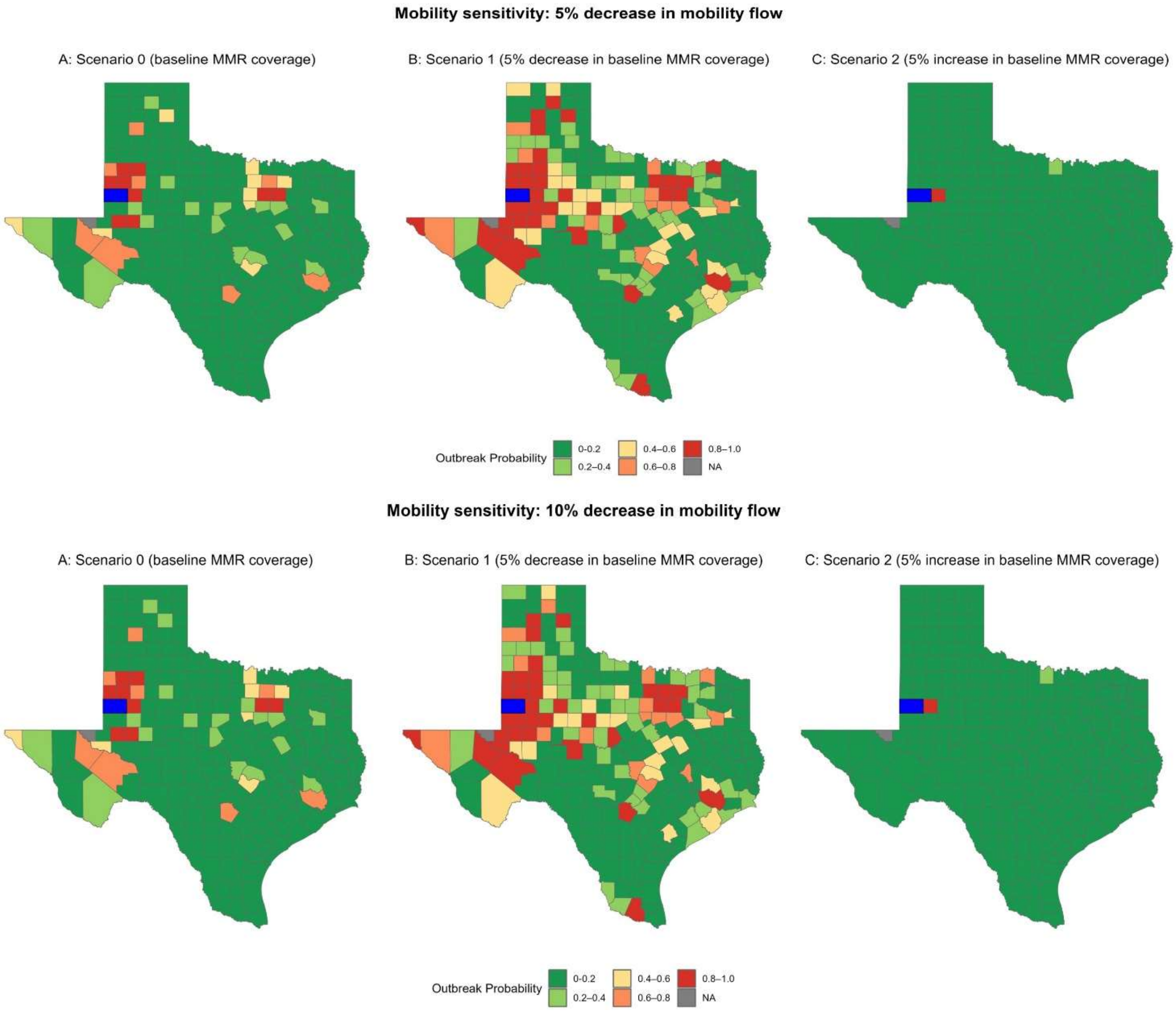

Supplementary Figure S2: Sensitivity analysis of the probability that a measles outbreak originating in Gaines County leads to major outbreaks in other Texas counties. Gaines County, the source of the outbreak, is denoted in blue. Here, all model parameters were kept at their Table 1 value, except mobility intensity which was decreased by 5% and 10%, respectively. A: Outbreak probability under the baseline MMR vaccine coverage. B: Outbreak probability under a 5% decrease in baseline MMR coverage in each county. C: Outbreak probability under a 5% increase in baseline MMR coverage in each county. The county shapefile was obtained from the US Census Bureau.

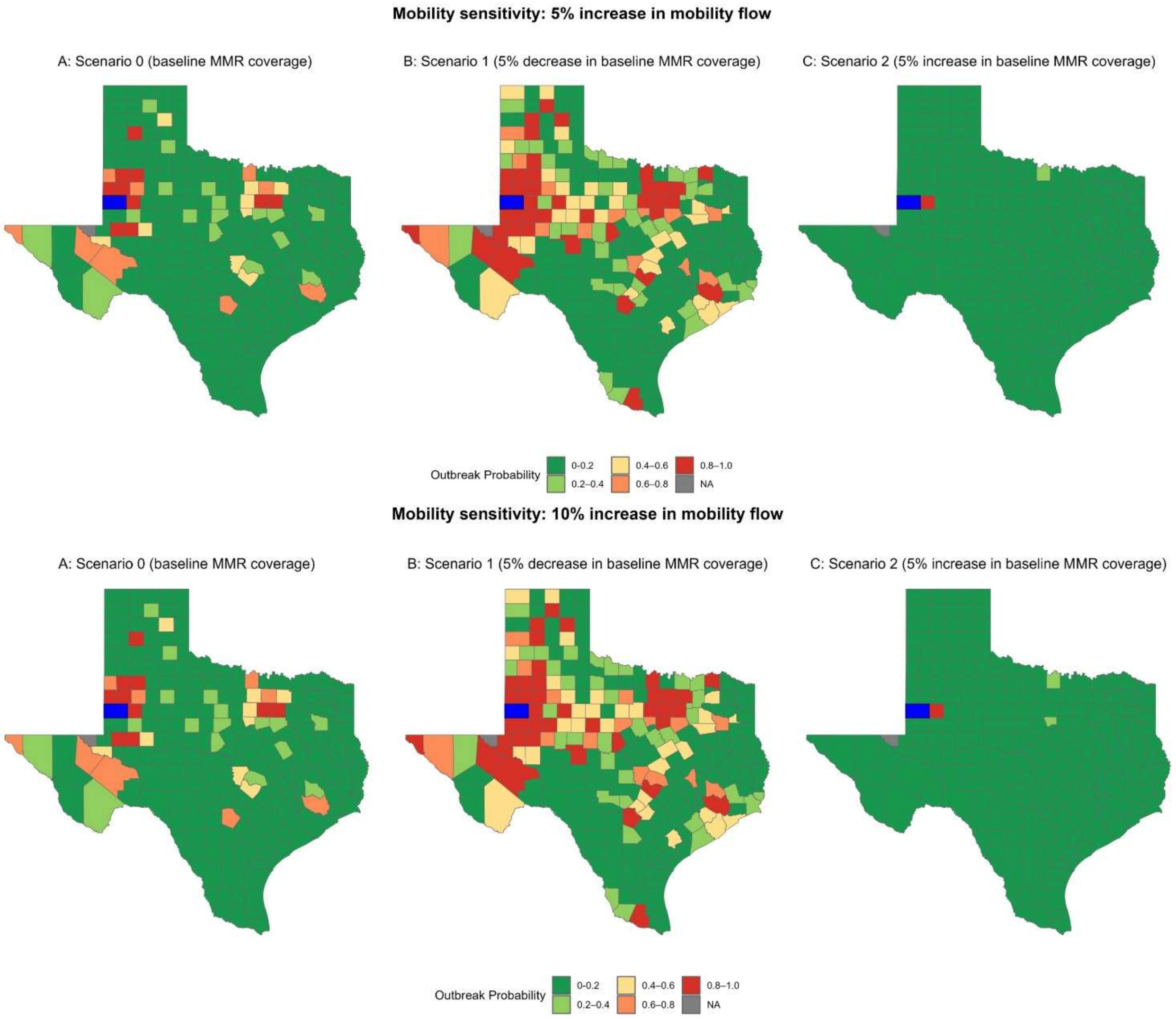

Supplementary Figure S3: Sensitivity analysis of the probability that a measles outbreak originating in Gaines County leads to major outbreaks in other Texas counties. Gaines County, the source of the outbreak, is denoted in blue. Here, all model parameters were kept at their Table 1 value, except mobility intensity which was increased by 5% and 10%, respectively. A: Outbreak probability under the baseline MMR vaccine coverage. B: Outbreak probability under a 5% decrease in baseline MMR coverage in each county. C: Outbreak probability under a 5% increase in baseline MMR coverage in each county. The county shapefile was obtained from the US Census Bureau.

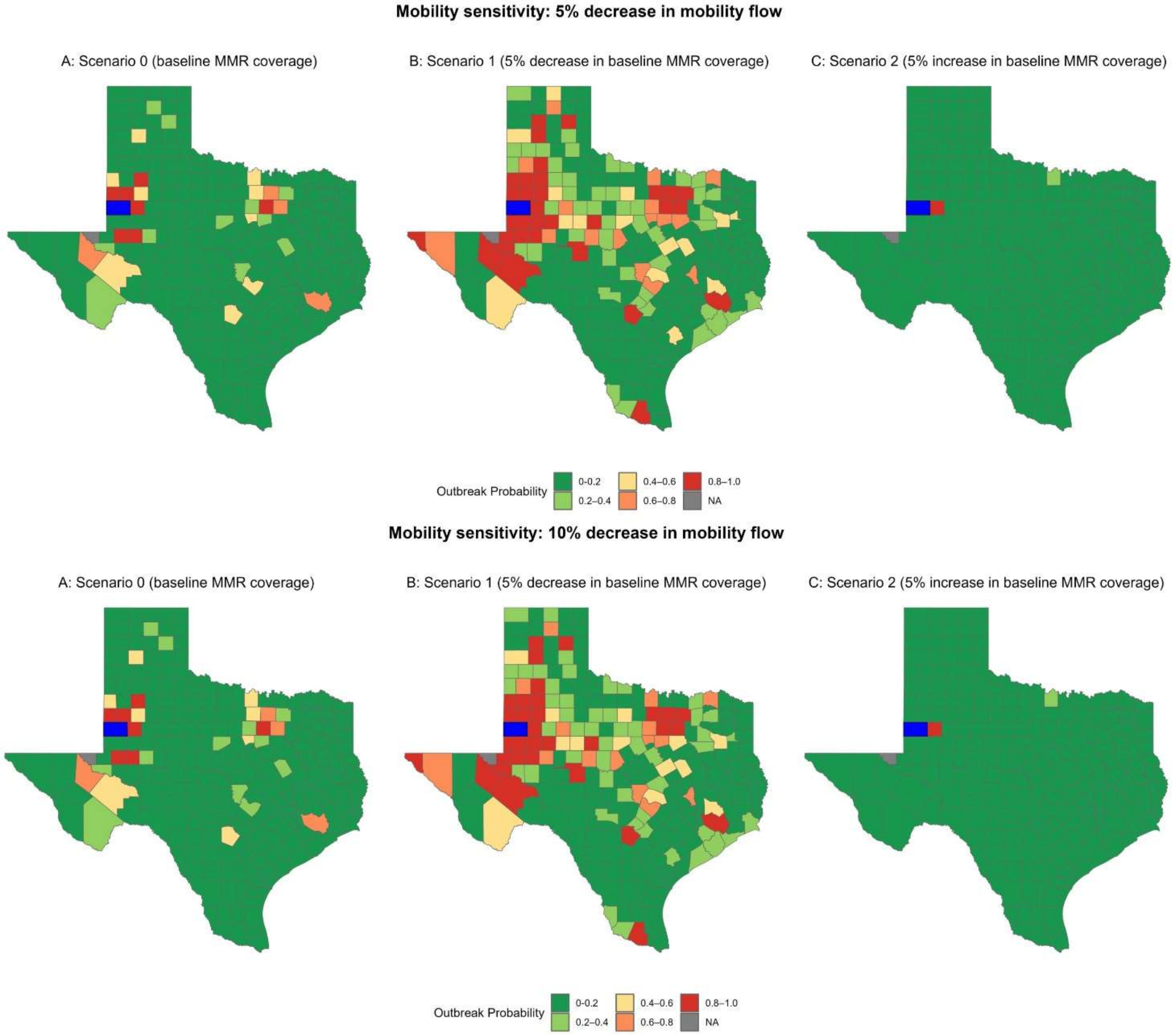

Supplementary Figure S4: Sensitivity analysis of the probability that a measles outbreak originating in Gaines County leads to major outbreaks in other Texas counties. Gaines County, the source of the outbreak, is denoted in blue. Here, all model parameters were kept at their Table 1 value, except R_0 value which was changed to 16 and mobility intensity which was decreased by 5% and 10%, respectively. A: Outbreak probability under the baseline MMR vaccine coverage. B: Outbreak probability under a 5% decrease in baseline MMR coverage in each county. C: Outbreak probability under a 5% increase in baseline MMR coverage in each county. The county shapefile was obtained from the US Census Bureau.

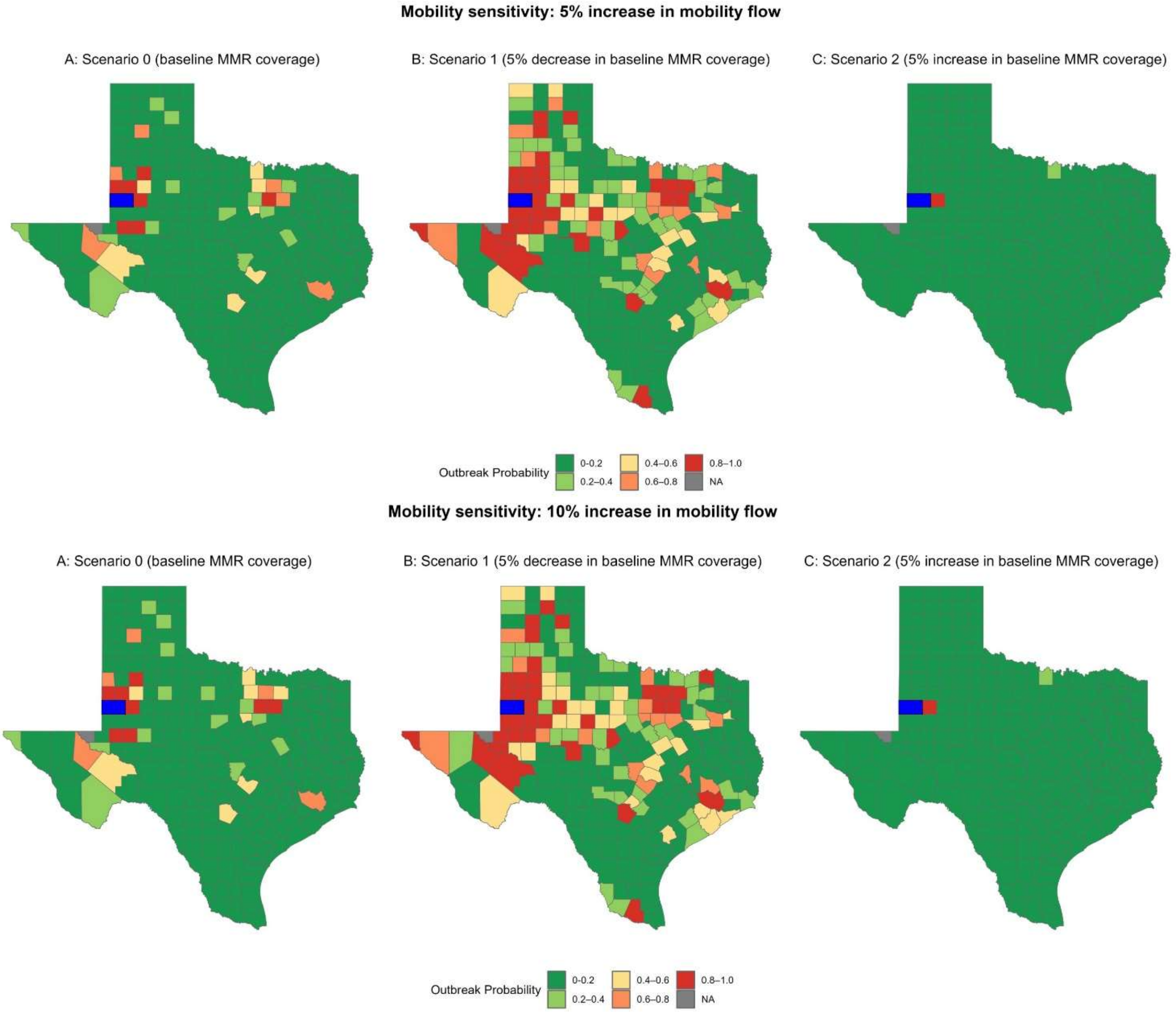

Supplementary Figure S5: Sensitivity analysis of the probability that a measles outbreak originating in Gaines County leads to major outbreaks in other Texas counties. Gaines County, the source of the outbreak, is denoted in blue. Here, all model parameters were kept at their Table 1 value, except R_0 value which was changed to 16 and mobility intensity which was increased by 5% and 10%, respectively. A: Outbreak probability under the baseline MMR vaccine coverage. B: Outbreak probability under a 5% decrease in baseline MMR coverage in each county. C: Outbreak probability under a 5% increase in baseline MMR coverage in each county. The county shapefile was obtained from the US Census Bureau.

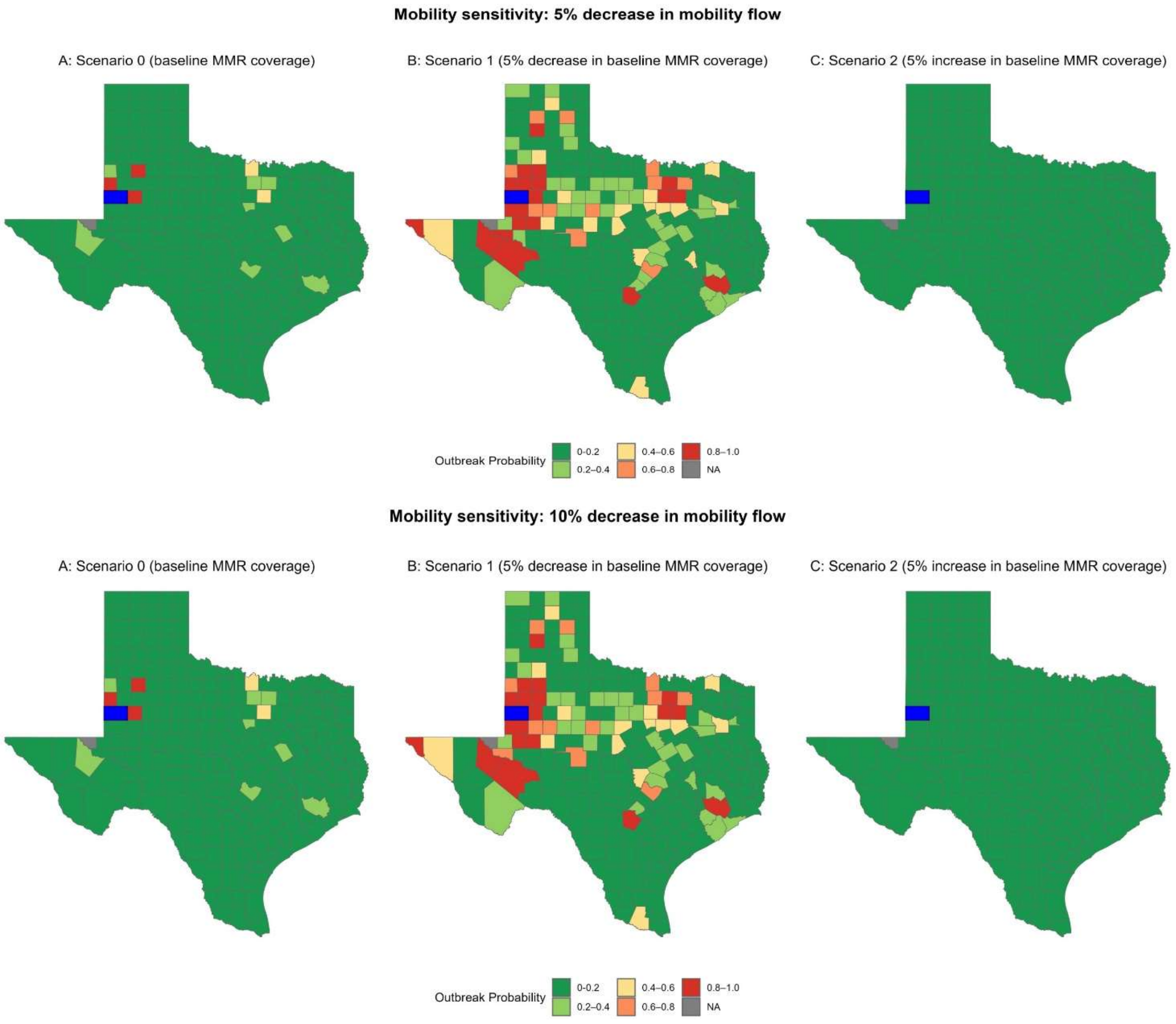

Supplementary Figure S6: Sensitivity analysis of the probability that a measles outbreak originating in Gaines County leads to major outbreaks in other Texas counties. Gaines County, the source of the outbreak, is denoted in blue. Here, all model parameters were kept at their Table 1 value, except R_0 value which was changed to 12 and mobility intensity which was decreased by 5% and 10%, respectively. A: Outbreak probability under the baseline MMR vaccine coverage. B: Outbreak probability under a 5% decrease in baseline MMR coverage in each county. C: Outbreak probability under a 5% increase in baseline MMR coverage in each county. The county shapefile was obtained from the US Census Bureau.

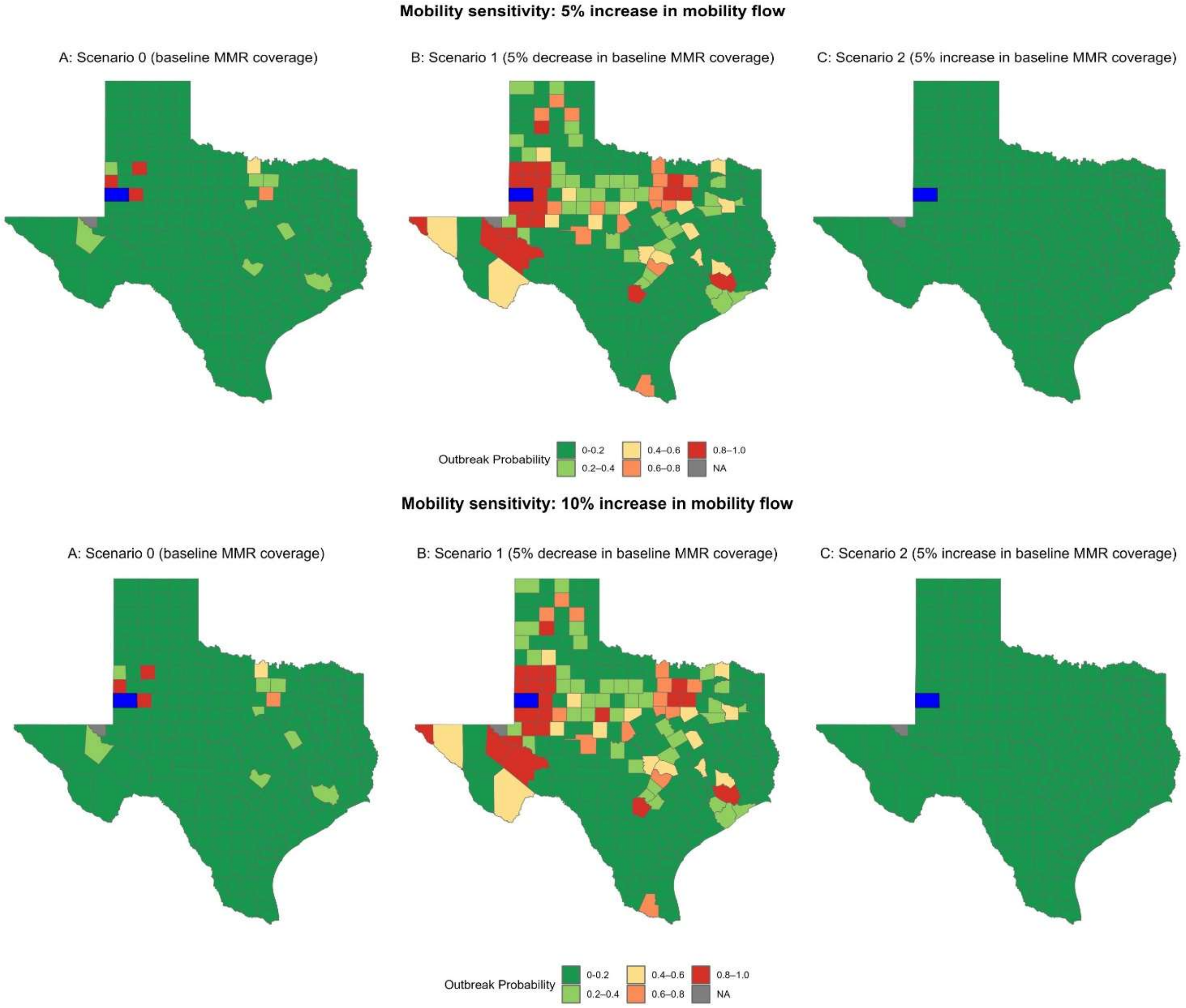

Supplementary Figure S7: Sensitivity analysis of the probability that a measles outbreak originating in Gaines County leads to major outbreaks in other Texas counties. Gaines County, the source of the outbreak, is denoted in blue. Here, all model parameters were kept at their Table 1 value, except R_0 value which was changed to 12 and mobility intensity which was increased by 5% and 10%, respectively. A: Outbreak probability under the baseline MMR vaccine coverage. B: Outbreak probability under a 5% decrease in baseline MMR coverage in each county. C: Outbreak probability under a 5% increase in baseline MMR coverage in each county. The county shapefile was obtained from the US Census Bureau.

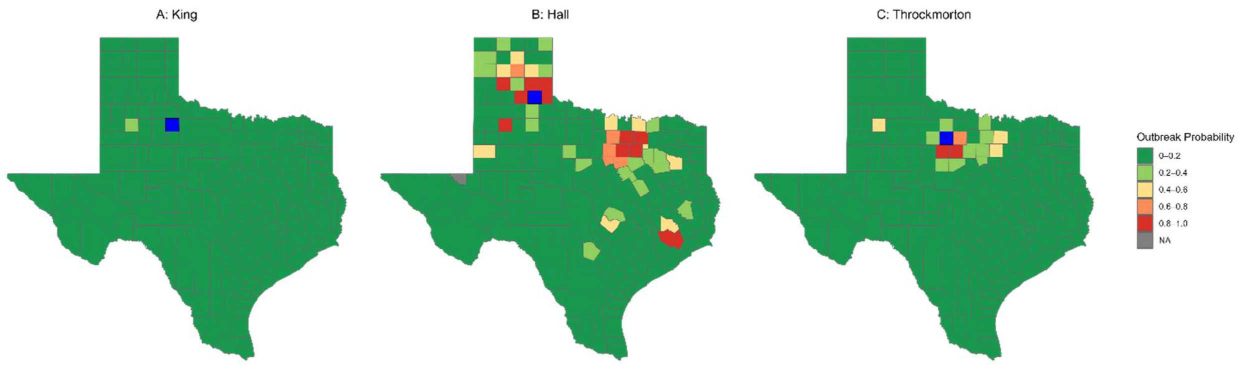

Supplementary Figure S8: Probability that a measles outbreak originating in King, Hall, and Throckmorton County leads to a major outbreak in other counties. Here, all model parameters were kept at their Table 1 value. **A:** Outbreak originating in King County; King County is denoted in blue. **B**: Outbreak originating in Hall County; Hall County is denoted in blue. **C**: Outbreak originating in Throckmorton County; Throckmorton County is denoted in blue.

Supplementary Table S1: Outbreak cases by county in West Texas as of August 12th, 2025.

County name	Confirmed cases
Andews	3
Atascosa	1
Bailey	2
Baxer	1
Borden	1
Brewster	1
Brown	1
Carson	1
Cochran	14
Collin	1
Dallan	7
Dawson	26

Eastland	2
Ector	12
El Paso	59
Erath	1
Fannin	4
Gaines	414
Garza	2
Hale	5
Hardeman	1
Hockley	7
Lamar	28
Lamb	1
Lubbock	52
Lynn	2
Martin	3
McLennan	9
Midland	6
Parmer	5
Potter	1
Randall	1
Reeves	2
Rokwall	1
Terry	60
Upshur	5
Yoakum	20
